# Do prescription rates of psychotropic drugs change over three years from nursing home admission?

**DOI:** 10.1186/s12877-021-02437-x

**Published:** 2021-09-16

**Authors:** Enrico Callegari, Jūratė Šaltytė Benth, Geir Selbæk, Cato Grønnerød, Sverre Bergh

**Affiliations:** 1 Østfold Hospital Trust, Sykehuset Østfold HF, postboks 300, 1714 Grålum, Norway; 2 Faculty of Medicine, University of Oslo, Oslo, Norway; 3 Institute of Clinical Medicine, Campus Ahus, University of Oslo, Oslo, Norway; 4 Research Centre for Age-related Functional Decline and Diseases, Innlandet Hospital Trust, Ottestad, Norway; 5 Health Services Research Unit, Akershus University Hospital, Lørenskog, Norway; 6 Norwegian National Advisory Unit on Ageing and Health, Vestfold Hospital Trust, Tønsberg, Norway; 7 Department of Geriatric Medicine, Oslo University Hospital, Oslo, Norway; 8 Faculty of Social Sciences, Department of Psychology, University of Oslo, Oslo, Norway

**Keywords:** Geriatric pharmacotherapy, Psychotropic drugs, Nursing homes

## Abstract

**Background:**

In this longitudinal study, we describe how psychotropic drugs (PTDs) are prescribed in nursing home (NH) patients from admission and over a 3-year period, to understand which clinical and environmental factors are associated with PTD prescription.

**Methods:**

We used data from the Resource Use and Disease Course in Dementia – Nursing Home (REDIC-NH) study, examining physical and mental health, dementia, and PTD prescription during a 3-year period from admission to a NH. Data were collected every six months. At baseline, we included 696 participants from 47 Norwegian NHs. We presented prevalence, incidence, and deprescribing rates of PTD prescriptions for each assessment point. We calculated the odds of receiving PTDs and used a generalized linear mixed model to analyze the variables associated with a change in odds throughout the 3-year period.

**Results:**

PTD prescriptions were frequent throughout the 3-year period. Antidepressants had the highest prescription rates (28.4%–42.2%). Every PTD category had the highest incidence rate between admission and six months, and antipsychotics had the highest values (49.4%). Deprescribing rates were comparable between assessment points. The odds of antipsychotic prescriptions were lower for older people (OR = 0.96, 95%CI:0.92–0.99, *p* = 0.023). People with more severe dementia had lower odds of being prescribed sedatives/hypnotics (OR = 0.89, 95%CI:0.85–0.94, *p* < 0.001).

**Conclusions:**

PTDs, particularly antidepressants, are widely prescribed over time to NH patients. Older patients are less likely to receive antipsychotics. A higher severity of dementia decreases the odds of being prescribed sedatives/hypnotics. Close attention should be paid to PTD prescriptions during long-term NH stay to avoid prolonged and excessive treatment with these types of drugs.

**Trial registration:**

ClinicalTrials.gov Identifier: NCT01920100.

## Background

Up to 84.3% of nursing home (NH) residents have dementia [1]. During the course of their NH stay, they often experience neuropsychiatric symptoms (NPS), in particular irritability, depression, and anxiety [2]. NPS are usually targeted with both pharmacological and non-pharmacological measures, where the latter is still considered first-line treatment [3].

Psychotropic drugs (PTDs) such as antidepressants, antipsychotics, and sedatives/hypnotics are primarily prescribed to treat psychiatric disorders, but are often prescribed in NH patients to treat NPS [4], despite recent Norwegian guidelines recommend to be cautious while prescribing these drugs [5]. In people with dementia, antidepressants are not very effective at treating depression [6], and atypical antipsychotics have a negligible effect on agitation and psychosis [7]. Non-patient related factors can also influence PTD prescriptions, such as staff-patient ratio and staff distress related to patients’ symptoms [8, 9], the knowledge gap among NH personnel about the related adverse effects of medication [10], communication education [11], and health care personnel’s positive belief or confidence in prescribing or discontinuing medication [12, 13]. Moreover, it can be challenging to monitor a drug therapy, as different screening tools for inappropriate prescribing may recommend different pharmacological measures [14].

The use of PTDs in older adults leads to a series of potential adverse effects that can worsen their physical and cognitive function [15]. Commonly-known adverse effects associated with short- or long-term PTD use, such as akathisia, agitation, aggression, and anxiety, can mislead the caregiver to think that NPS are worsening, leading to a further increase in PTD dosages [16]. In addition, up to 86% of NH residents are exposed to polypharmacy (≥5 concomitant drugs) [17], increasing the risk of several adverse effects, morbidity, mortality, as well as inappropriate prescribing [18].

Detecting an inappropriate therapy at an early stage of NH stay might help physicians avoid later complications. A vast body of literature describes PTD prescriptions in NHs. Most of the studies have a cross-sectional nature and vary in their methodological approaches, which makes it challenging to compare results [8, 19–21]. The longitudinal aspects of PTD prescriptions are important in order to find possible explanations behind treatment decisions over time. A recent study has shown frequent and persistent use of PTDs in Norwegian NHs during a 72-months follow-up [22]. The assessment of patients from admission is also particularly relevant, as NH transitions may worsen the residents’ psychiatric symptoms and their perceived quality of life [23], possibly leading physicians to initiate a pharmacological treatment during this transition. Very few longitudinal studies have described PTD prescription rates in NH residents from admission [24–26], and even fewer have described PTD prescriptions in relation to physical, cognitive, psychological, and environmental factors [27, 28]. None have presented a comprehensive analysis of systematic clinical factors, NPS, and environmental factors and their association with PTD prescriptions.

A recent study based on the same data material used in this paper has explored which clinical factors at admission could predict changes in PTD prescriptions six months after NH admission [29]. Besides a general increase in prescription of all the major PTDs during the first six months, higher affective subsyndrome scores for the Neuropsychiatric Inventory 12-item nursing home version (NPI-NH) were associated with a higher odds of prescribing antidepressants, sedatives, and hypnotics at admission and six months later [29, 30].

The aim of this paper is to investigate the course of PTD prescription in NH patients, focusing on prevalence, incidence and deprescribing rates, and their relationship to clinical and environmental factors, during a three-year follow-up from admission to NHs.

## Methods

We used data from the Resource Use and Disease Course in Dementia - Nursing Home (REDIC-NH) study, designed to follow NH residents from admission until death [31]. At baseline (BL), 696 patients admitted to 47 Norwegian NHs were included.

**Fig. 1:**
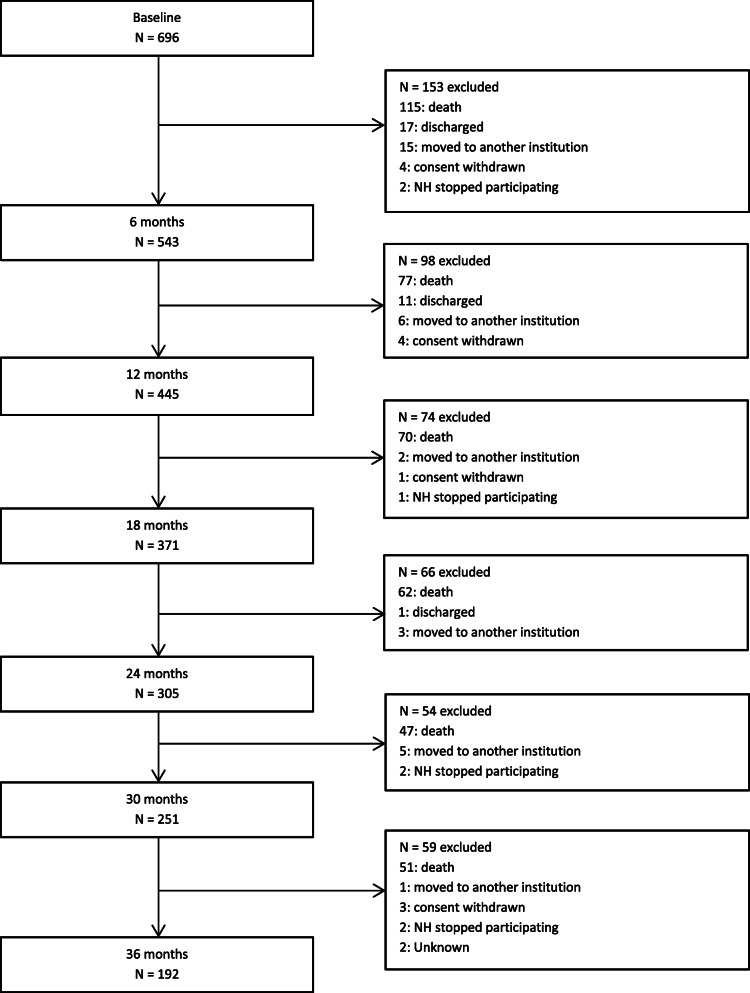
Flow chart: selection of patients for analysis

Among 47 recruited NHs, only 38 NHs collected information (gender and age) on eligible patients not included in the study. As described by Røen et al. (2017), in these 38 NHs 1331 patients were eligible for inclusion, 724 patients were excluded, and 607 were included [31]. For the remaining nine NHs, we do not, unfortunately, have information about not-inclusion, but the nine NHs included 89 patients giving a total of 696 included patients in the study. The NHs, representing small and large facilities, were situated in urban and rural areas in four Norwegian counties [31]. BL assessments were registered between March 2012 and November 2014, and the participants were further assessed every six months until death or until 3-year NH-stay. To be included at BL, patients had to be at least 65 years old or younger than 65 years with established dementia, had to have a life expectancy > 6 weeks and an expected NH stay of > 4 weeks. The flow chart for the sample inclusion, together with attrition causes between each assessment point, are presented in Fig. 1.

Demographic data were registered at BL. Dementia at BL was diagnosed by SB and GS according to ICD-10 criteria, based on all collected data. At each assessment point, NH characteristics and daily medication use according to the ATC system were registered. Data regarding medication “as needed” was not recorded. PTDs were grouped as follow: antidepressants (N06A), antipsychotics (N05A, consisting of typical and atypical antipsychotics, except lithium), anxiolytics (N05B), sedatives and hypnotics (N05C), and antidementia drugs (N06D, consisting of cholinesterase inhibitors and memantine). Validated instruments were used to assess dementia severity (the Clinical Dementia Rating (CDR) scale) [32], level of functioning (the Physical Self-Maintenance Scale - PSMS) [33], NPS (the Neuropsychiatric Inventory 12-item nursing home version – NPI-NH - and the Cornell Scale for Depression in Dementia - CSDD) [30, 34], physical function (the General Medical Health Rating (GMHR) scale and the Charlson Comorbidity Index) [35, 36], and quality of life (the Quality of Life (QoL) in Late-Stage Dementia (QUALID) scale) [37].

### Statistical analyses

Demographic, clinical, and environmental characteristics at BL are presented as means and standard deviations (SDs) for continuous variables, and frequencies and percentages for categorical variables. We calculated the prevalence, incidence rate and deprescribing rate of prescription for any PTD as well as for each PTD subgroup (antidepressants, antipsychotics, anxiolytics, sedatives and hypnotics, and antidementia drugs). We defined prevalence as the proportion of patients prescribed a particular PTD at each assessment point. Incidence rate / deprescribing rate was defined as the proportion of patients prescribed / deprescribed a particular PTD at one assessment point relative to the number of patients not prescribed / prescribed the same PTD at the previous assessment point. We present the total number of medications and the total number of PTDs as mean and SD, the numbers for the whole cohort, as well as stratified by dementia diagnosis.

We estimated an unadjusted generalized linear mixed model with second-order time component to assess a possible non-linear trend in odds for use of antidepressants, antipsychotics, anxiolytics, sedatives and hypnotics, and antidementia drugs. Pre-chosen covariates assessed at baseline or simultaneously with drug use covariates, one at a time, were included into the model as additional fixed effects together with the interaction term between the covariate and time. Finally, we estimated an adjusted model with time, all covariates and interactions included. We applied Bayesian Information Criterion (smaller values means better model) to eliminate excessive interactions. A significant interaction implies that a covariate is significantly associated with change in odds over time. All models included random effects for patients nested within NHs. The unadjusted time trend is illustrated graphically as odds of being prescribed a particular PTD at each assessment point with 95% confidence intervals (CI). The associations between covariates and prescription of a particular PTD were tabulated as odds ratios (OR) and 95% CI whenever interaction was absent. Regression coefficients and standard errors (SEs) are presented for covariates included in the interactions. For easier interpretation, these results are also illustrated graphically. All tests were two-sided and results with *p*-values ≤0.05 were considered statistically significant.

Most covariates had some missing values. For cases with fewer than 50% missing values on items of a particular scale (CDR, CSDD, PSMS, QoL, and NPS scores), we imputed missing values for each item separately by drawing a random number from its empirical distribution. For the Charlson Comorbidity Index, we substituted missing values with zero.

We used IBM® SPSS® Statistics version 26® and SAS Institute Inc.® SAS® version 9.4 statistical software for the analyses.

## Results

At BL, 696 patients were included. The majority had dementia (83.8%), were female (64.1%), had a fair/poor physical health (52.4%), and lived in a regular NH unit (55.3%) (Table 1).

**Table 1:** Demographic and clinical data of patients at nursing home admission, *N* = 696

Variable	No dementia *N* = 113	Dementia *N* = 583	Total *N* = 696
Age			
N	113	580	693
Mean (SD)	86.4 (7.0)	84.0 (7.5)	84.4 (7.5)
Gender, female			
n/N (%)	70/113 (61.9)	376/583 (64.5)	446/696 (64.1)
GMHR			
Poor/Fair, n/N (%)	69/109 (63.3)	280/557 (50.3)	349/666 (52.4)
Good/Excellent, n/N (%)	40/109 (36.7)	277/557 (49.7)	317/666 (47.6)
Charlson Comorbidity Index			
N	104	525	629
Mean (SD)	3.5 (2.8)	2.8 (2.1)	2.9 (2.3)
PSMS			
N	112	582	694
Mean (SD)	1.5 (1.3)	1.5 (1.3)	1.5 (1.3)
MMSE			
N	104	516	620
Mean (SD)	22.5 (5.6)	14.8 (5.5)	16.1 (6.2)
CDR sum of boxes			
N	111	578	689
Mean (SD)	5.3 (4.2)	11.3 (3.6)	10.3 (4.3)
CSDD			
N	109	551	660
Mean (SD)	5.7 (4.7)	6.7 (5.3)	6.5 (5.2)
NPI total			
N	112	573	685
Mean (SD)	9.2 (12.5)	15.4 (17.5)	14.4 (17.0)
NPI-agitation^a^			
N	112	580	692
Mean (SD)	2.0 (4.8)	4.5 (7.3)	4.1 (7.0)
NPI-psychosis^a^			
N	112	570	682
Mean (SD)	0.7 (2.3)	1.9 (4.2)	1.7 (4.0)
NPI-affective^a^			
N	112	577	689
Mean (SD)	2.8 (4.6)	3.9 (5.9)	3.7 (5.7)
NPI-caregivers			
N	112	581	693
Mean (SD)	3.4 (5.0)	6.0 (7.4)	5.5 (7.2)
NPI-apathy			
N	112	574	686
Mean (SD)	1.1 (2.7)	1.4 (2.8)	1.3 (2.8)
QUALID			
N	112	580	692
Mean (SD)	19.3 (6.9)	20.0 (7.2)	19.9 (7.2)
MOBID-II			
N	110	557	667
Mean (SD)	2.8 (2.4)	2.0 (2.1)	2.1 (2.1)
Type of unit			
Regular unit, n/N (%)	82/113 (72.6)	303/583 (52.0)	385/696 (55.3)
Special care unit, n/N (%)	10/113 (8.8)	216/583 (37.0)	226/696 (32.5)
Respite and rehabilitation unit, n/N (%)	21/113 (18.6)	64/583 (11.0)	85/696 (12.2)
Number of patients per unit			
N	113	581	694
Mean (SD)	14.6 (7.1)	11.4 (5.8)	11.9 (6.1)
Number of staff members per unit working dayshift			
N	113	582	695
Mean (SD)	4.2 (2.2)	3.6 (1.9)	3.7 (2.0)
Number of hours a physician is present per unit			
N	102	467	569
Mean (SD)	4.7 (4.5)	3.7 (4.7)	3.9 (4.6)

*SD* Standard deviation, *GMHR* General Medical Health Rating Scale, *PSMS* Physical Self-Maintenance Scale, *MMSE* Mini-Mental Status Evaluation, *CDR* Clinical Dementia Rating scale, *CSDD* Cornell Scale for Depression in Dementia, *NPI* Neuropsychiatric Inventory, *QUALID* Quality of Life in Late-Stage Dementia, *MOBID-II* Mobilization-Observation-Behaviour-Intensity-Dementia Pain Scale

^a^ NPI-subsyndromes are calculated as the sum of the following items: NPI-Agitation = Agitation + Disinhibition + Irritability, NPI-Psychosis = Delusions + Hallucinations, NPI-Affective = Depression + Anxiety

**Fig. 2:**
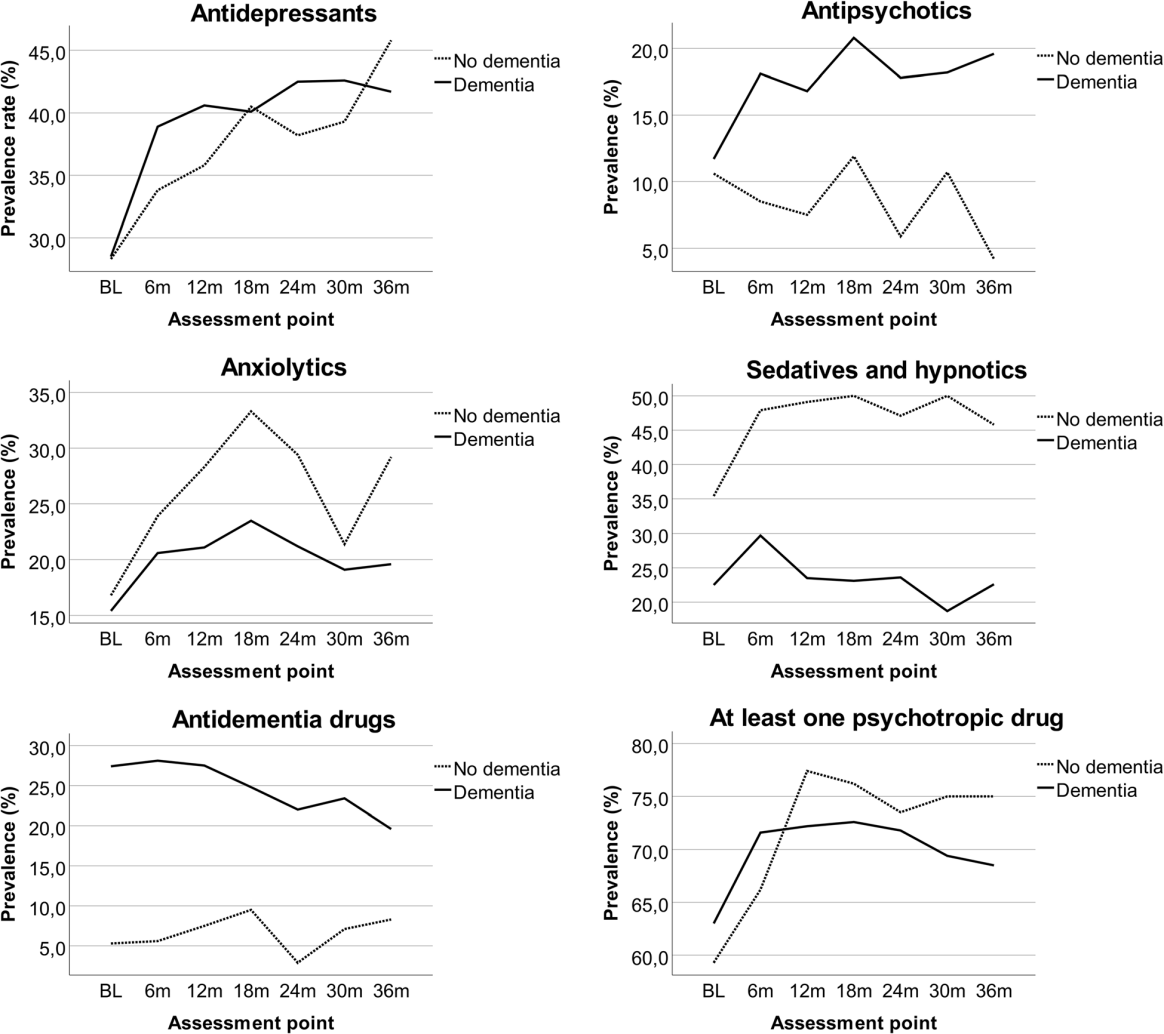
Prevalence of psychotropic drugs prescription between baseline (BL) and 36 months (36m)

**Fig. 3:**
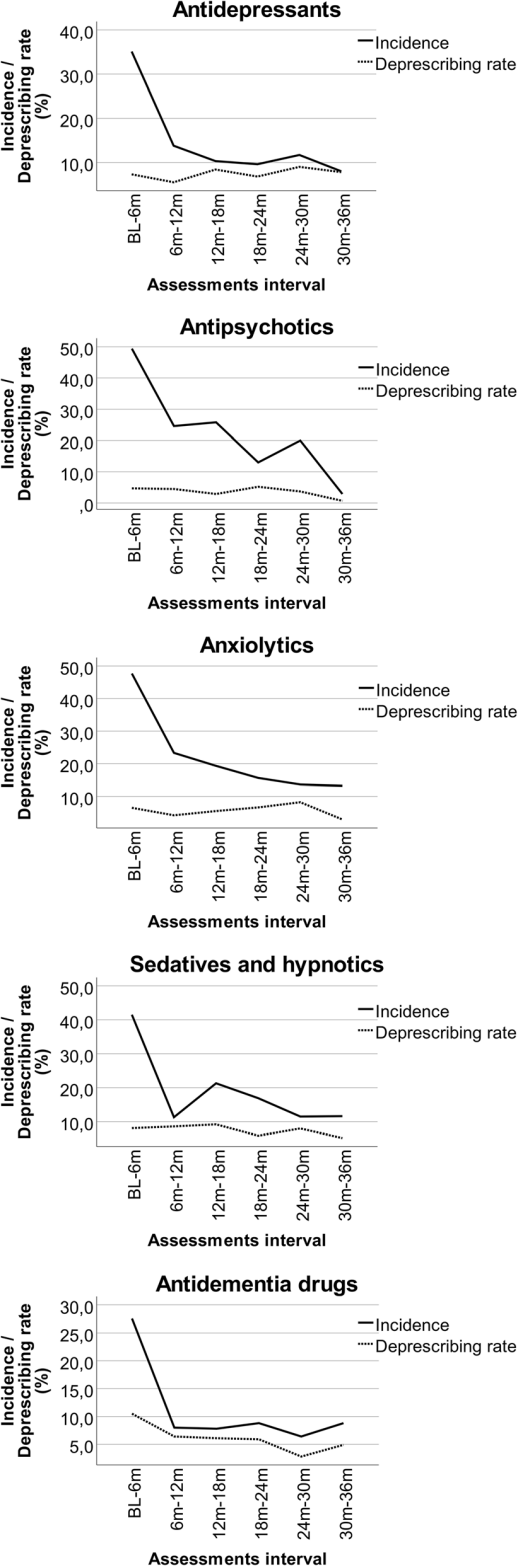
Incidence and deprescribing rates of psychotropic drugs between baseline (BL) and 36 months (36m)

Prevalence, incidence and deprescribing rates for the major PTD categories are presented Table 2. Selected results are illustrated in Figs. 2 and 3.

**Table 2:** Prevalence, incidence, and deprescribing rates of psychotropic drugs: numbers are percentages

Prevalence																					
	BL *N* = 113 (D-); 583 (D+); 696 (T)			6m *N* = 71 (D-); 437 (D+); 508 (T)			12m *N* = 53 (D-); 374 (D+); 427 (T)			18m *N* = 42 (D-); 307 (D+); 349 (T)			24m *N* = 34 (D-); 259 (D+); 293 (T)			30m *N* = 28 (D-); 209 (D+); 237 (T)			36m *N* = 24 (D-); 168 (D+); 192 (T)		
Drug category	D-	D+	T	D-	D+	T	D-	D+	T	D-	D+	T	D-	D+	T	D-	D+	T	D-	D+	T
Antidepressants	28.3	28.5	28.4	33.8	38.9	38.2	35.8	40.6	40.0	40.5	40.1	40.1	38.2	42.5	42.0	39.3	42.6	42.2	45.8	41.7	42.2
Atypical antipsychotics	6.2	7.0	6.9	4.2	13.7	12.4	1.9	12.6	11.2	4.8	16.0	14.6	2.9	14.3	13.0	7.1	14.8	13.9	0	16.7	14.6
Typical antipsychotics	5.3	4.8	4.9	5.6	4.6	4.7	5.7	4.3	4.4	7.1	4.9	5.2	2.9	3.9	3.8	3.6	3.8	3.8	4.2	3.0	3.1
Any antipsychotic	10.6	11.7	11.5	8.5	18.1	16.7	7.5	16.8	15.7	11.9	20.8	19.8	5.9	17.8	16.4	10.7	18.2	17.3	4.2	19.6	17.7
Anxiolytics	16.8	15.4	15.7	23.9	20.6	21.1	28.3	21.1	22.0	33.3	23.5	24.6	29.4	21.2	22.2	21.4	19.1	19.4	29.2	19.6	20.8
Sedatives and hypnotics	35.4	22.5	24.6	47.9	29.7	32.3	49.1	23.5	26.7	50.0	23.1	26.4	47.1	23.6	26.3	50.0	18.7	22.4	45.8	22.6	25.5
Antidementia drugs	5.3	27.4	23.9	5.6	28.1	25.0	7.5	27.5	25.1	9.5	24.8	22.9	2.9	22.0	19.8	7.1	23.4	21.5	8.3	19.6	18.2
Cholinesterase inhibitors	2.7	20.2	17.4	4.2	19.5	17.3	5.7	18.4	16.9	7.1	16.3	15.1	2.9	13.1	11.9	3.6	13.9	12.7	4.2	11.9	10.9
At least one PTD^a^	59.3	63.0	62.4	66.2	71.6	70.9	77.4	72.2	72.8	76.2	72.6	73.1	73.5	71.8	72.0	75.0	69.4	70.0	75.0	68.5	69.3
Mean (SD)																					
Total medication - mean	7.3	5.7	6.0	8.2	6.2	6.5	7.5	6.0	6.2	7.2	5.9	6.1	7.3	6.2	6.3	7.2	6.3	6.4	7.8	6.3	6.5
(SD)	(3.5)	(3.1)	(3.2)	(3.5)	(3.0)	(3.1)	(3.4)	(3.0)	(3.1)	(3.6)	(3.3)	(3.3)	(3.5)	(3.3)	(3.3)	(3.9)	(3.3)	(3.4)	(3.9)	(3.7)	(3.7)
Total PTD^a^ − mean	1.1	1.1	1.2	1.3	1.5	1.5	1.4	1.4	1.4	1.6	1.4	1.5	1.4	1.4	1.4	1.4	1.3	1.4	1.5	1.4	1.4
(SD)	(1.2)	(1.2)	(1.2)	(1.3)	(1.5)	(1.3)	(1.2)	(1.2)	(1.2)	(1.3)	(1.3)	(1.3)	(1.1)	(1.2)	(1.2)	(1.2)	(1.2)	(1.2)	(1.4)	(1.2)	(1.3)
Incidence^b^																					
	BL-6m N = 71 (D-); 437 (D+); 508 (T)			6m–12m *N* = 51 (D-); 346 (D+); 397 (T)			12m–18m *N* = 40 (D-); 298 (D+); 338 (T)			18m–24m N = 30 (D-); 246 (D+); 276 (T)			24m–30m *N* = 27 (D-); 200 (D+); 227 (T)			30m–36m *N* = 22 (D-); 156 (D+); 178 (T)					
Drug category	D-	D+	T	D-	D+	T	D-	D+	T	D-	D+	T	D-	D+	T	D-	D+	T			
Antidepressants	37.5	34.7	35.1	16.7	13.4	13.8	17.6	9.2	10.3	9.1	9.7	9.6	10.0	11.9	11.7	22.2	6.1	8.0			
Atypical antipsychotics	33.3	60.0	58.7	0	28.9	28.3	0	23.4	22.9	100	16.7	18.9	100	20.0	25.0	0	7.1	7.1			
Typical antipsychotic	25.0	50.0	45.8	0	37.5	31.6	33.3	40.0	38.9	0	11.1	10.0	0	0	0	0	0	0			
Any antipsychotic	33.3	50.6	49.4	0	26.2	24.6	25.0	25.8	25.8	50.0	11.4	13.0	66.7	16.2	20.0	0	3.0	2.9			
Anxiolytics	41.2	48.9	47.7	28.6	22.2	23.3	28.6	17.4	19.3	0	18.5	15.6	0	15.4	13.6	16.7	12.5	13.2			
Sedatives and hypnotics	29.4	44.6	41.5	4.2	13.4	11.3	14.3	23.5	21.3	21.4	15.8	16.9	7.7	12.8	11.5	0	15.2	11.6			
Antidementia drugs	50.0	26.8	27.6	0	8.3	8.0	0	8.1	7.8	0	8.9	8.8	0	6.5	6.4	0	9.4	8.8			
Cholinesterase inhibitors	66.7	25.9	27.3	0	10.8	10.3	0	8.2	7.8	0	9.1	8.8	0	7.7	7.4	0	10.0	9.5			
Deprescribing rates^b^																					
	BL-6m N = 71 (D-); 437 (D+); 508 (T)			6m–12m N = 51 (D-); 346 (D+); 397 (T)			12m–18m N = 40 (D-); 298 (D+); 338 (T)			18m–24m N = 30 (D-); 246 (D+) 276 (T)			24m–30m N = 27 (D-); 200 (D+); 227 (T)			30m–36m N = 22 (D-); 156 (D+); 178 (T)					
Drug category	D-	D+	T	D-	D+	T	D-	D+	T	D-	D+	T	D-	D+	T	D-	D+	T			
Antidepressants	8.5	7.1	7.3	6.1	5.4	5.5	4.3	8.9	8.4	5.3	7.0	6.8	11.8	8.6	9.0	7.7	7.8	7.8			
Atypical antipsychotics	1.5	2.4	2.2	0	4.0	3.4	0	2.8	2.4	0	4.8	4.2	0	4.1	3.6	0	0.8	0.7			
Typical antipsychotics	1.5	3.6	3.3	0	2.1	1.9	0	0.7	0.6	3.4	0.8	1.1	0	0	0	0	0.7	0.6			
Any antipsychotic	3.1	5.0	4.7	0	5.3	4.5	0	3.4	2.9	3.6	5.4	5.2	0	4.3	3.7	0	0.8	0.7			
Anxiolytics	1.9	7.2	6.5	10.8	3.3	4.2	0	6.1	5.5	5.0	6.8	6.6	13.6	7.5	8.2	0	3.2	2.9			
Sedatives and hypnotics	13.5	7.5	8.1	14.8	8.0	8.6	21.1	8.3	9.2	18.8	4.8	5.9	14.3	7.5	8.0	16.7	4.1	5.2			
Antidementia drugs	1.5	12.4	10.5	0	7.6	6.4	0	7.1	6.1	0	6.8	5.9	0	3.2	2.8	0	5.6	4.9			
Cholinesterase inhibitors	1.5	8.2	7.1	0	6.4	5.5	0	5.2	4.5	0	3.8	3.3	0	2.3	2.0	0	4.4	3.8			

D+: dementia at baseline; D-: no dementia at baseline; T: total

^a^ PTD: psychotropic drugs

^b^ Inclusion of cases with observations at both assessment points

**Fig. 4:**
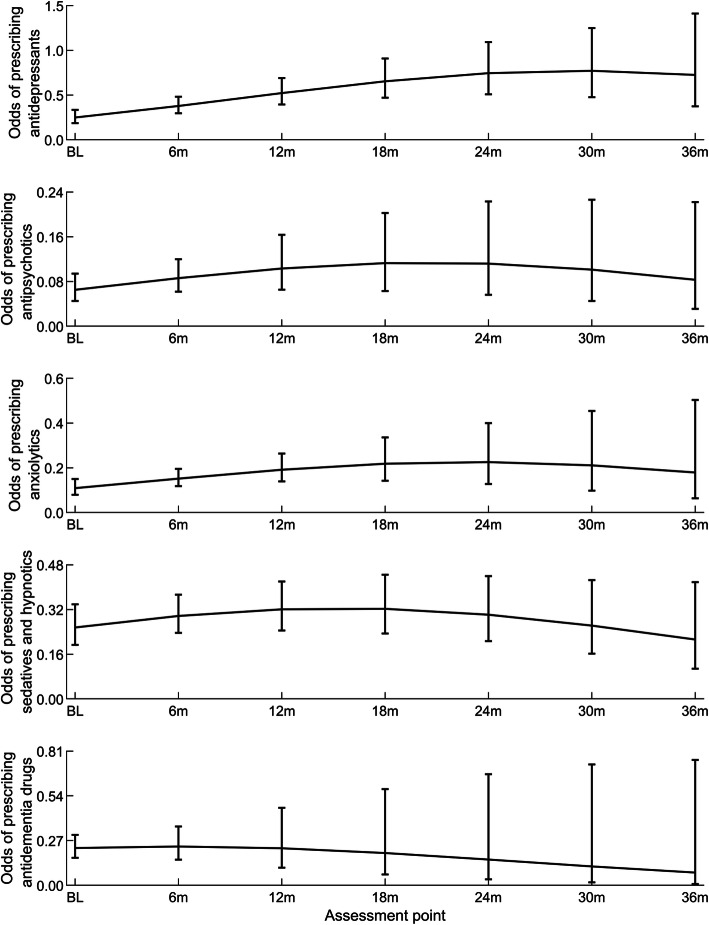
Graphical representation of the unadjusted time trends for the odds of prescribing psychotropic drugs

According to unadjusted generalized linear mixed models, there was a significant non-linear time trend in odds of prescribing antidepressants and anxiolytics, but not for antipsychotics, sedatives and hypnotics, or antidementia drugs (Fig. 4).

**Fig. 5:**
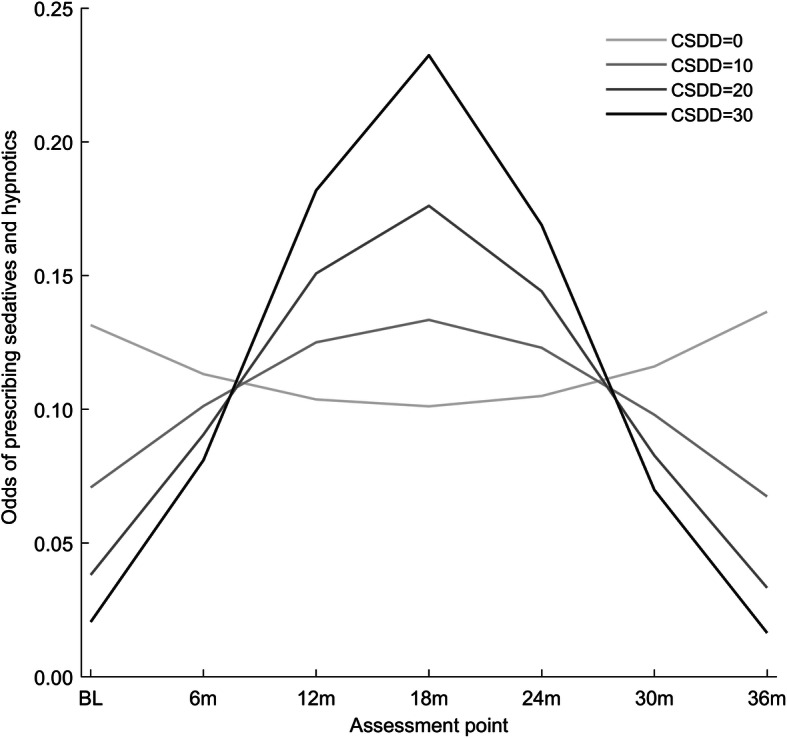
Interaction between CSDD^†^ score and change in odds of prescribing sedatives and hypnotics: graphical representation. Legends: ^†^ CSDD: Cornell Scale for Depression in Dementia

Table 3 presents the results of adjusted generalized linear mixed models. Time trend in odds of prescribing certain PTDs remained nearly unchanged after adjustment for covariates. None of the covariates were associated with *change* in odds over time for the five assessed PTD categories (non-significant interactions between covariates and time), except for CSDD, which was significantly associated with change in odds of prescribing sedatives and hypnotics. For CSDD scores < 8, the change in odds of prescribing sedatives and hypnotics was not significantly associated with CSDD. For CSDD scores > 8, the increasing CSDD score was associated with higher odds of prescribing sedatives and hypnotics from BL to 18m, and decreased odds of prescribing sedatives and hypnotics from 18m to 36m (Fig. 5).

**Table 3:** Results from the *generalized linear mixed model*^a^ for antidepressants, antipsychotics, anxiolytics, sedatives/hypnotics, and antidementia drugs

Covariate	Antidepressants		Antipsychotics		Anxiolytics		Sedatives and hypnotics		Antidementia drugs	
	Reg. coeff. (SE)	*p*-value	Reg. coeff. (SE)	*p*-value	Reg. coeff. (SE)	*p*-value	Reg. coeff. (SE)	*p*-value	Reg. coeff. (SE)	*p*-value
Time	**0.07 (0.02)**	**< 0.001**	0.04 (0.03)	0.157	**0.06 (0.02)**	**0.005**	−0.03 (0.03)	0.358	0.01 (0.02)	0.561
Time*Time	**−0.001 (0.0006)**	**0.023**	−0.001 (0.0008)	0.150	**−0.001 (0.0006)**	**0.029**	0.0009 (0.001)	0.374	−0.001 (0.0007)	0.135
	OR (95% CI)	p-value	OR (95% CI)	p-value	OR (95% CI)	p-value	OR (95% CI)	p-value	OR (95% CI)	p-value
Age	**0.93 (0.90–0.97)**	**< 0.001**	**0.96 (0.92–0.99)**	**0.023**	0.98 (0.95–1.01)	0.172	1.02 (0.99–1.05)	0.213	0.97 (0.93–1.00)	0.062
Gender, female	**2.09 (1.26–3.47)**	**0.005**	1.17 (0.64–2.15)	0.608	0.99 (0.59–1.65)	0.961	0.90 (0.56–1.45)	0.676	1.01 (0.59–1.74)	0.973
Charlson Comorbidity Index	1.02 (0.91–1.14)	0.748	0.96 (0.60–1.53)	0.097	0.97 (0.87–1.09)	0.640	0.99 (0.89–1.09)	0.810	**0.86 (0.75–0.98)**	**0.023**
GMHR, Poor/Fair	1.04 (0.73–1.50)	0.824	0.96 (0.60–1.53)	0.851	1.17 (0.79–1.73)	0.440	0.74 (0.52–1.06)	0.104	0.78 (0.52–1.15)	0.207
PSMS	0.85 (0.72–1.01)	0.070	0.90 (0.72–1.13)	0.358	0.99 (0.82–1.19)	0.890	0.85 (0.72–1.01)	0.059	1.16 (0.96–1.39)	0.121
CDR sob	0.98 (0.93–1.04)	0.519	1.02 (0.95–1.09)	0.597	0.98 (0.93–1.04)	0.464	**0.89 (0.85–0.94)**	**< 0.001**	1.03 (0.97–1.09)	0.337
CSDD	**1.05 (1.00–1.10)**	**0.045**	0.98 (0.93–1.04)	0.590	1.02 (0.97–1.07)	0.411	**−0.06 (0.03)**^**b**^	**0.040**	0.95 (0.90–1.00)	0.075
CSDD * Time							**0.01 (0.004)**^**b**^	**0.010**		
CSDD * Time * Time							**−0.0003 (0.0001)**^**b**^	**0.014**		
NPI-agitation	1.00 (0.96–1.03)	0.868	1.00 (0.96–1.04)	0.980	1.01 (0.98–1.04)	0.626	0.99 (0.96–1.02)	0.446	0.97 (0.94–1.01)	0.136
NPI-psychosis	0.98 (0.93–1.03)	0.391	**1.11 (1.05–1.17)**	**< 0.001**	1.02 (0.97–1.07)	0.515	0.98 (0.93–1.04)	0.549	1.04 (0.98–1.09)	0.212
NPI-affective	**1.09 (1.04–1.14)**	**< 0.001**	1.03 (0.98–1.08)	0.227	**1.05 (1.01–1.10)**	**0.026**	1.04 (0.99–1.08)	0.083	1.00 (0.96–1.05)	0.929
NPI-caregivers	1.00 (0.95–1.04)	0.851	1.00 (0.95–1.05)	0.998	0.99 (0.94–1.03)	0.581	1.03 (0.98–1.08)	0.201	1.03 (0.98–1.09)	0.222
QUALID	0.97 (0.94–1.01)	0.094	1.01 (0.97–1.06)	0.541	1.02 (0.98–1.05)	0.416	1.03 (0.99–1.06)	0.134	1.00 (0.96–1.04)	0.827
NPI-apathy	1.04 (0.98–1.11)	0.175	1.00 (0.93–1.08)	0.984	0.98 (0.92–1.05)	0.657	1.03 (0.97–1.10)	0.265	**0.93 (0.86–1.00)**	**0.039**
Type of unit: special care unit	0.81 (0.50–1.30)	0.379	1.32 (0.75–2.34)	0.332	1.58 (0.98–2.57)	0.062	1.54 (0.96–2.45)	0.071	**1.78 (1.09–2.90)**	**0.021**
No. patients/unit	0.97 (0.93–1.00)	0.080	1.00 (0.95–1.05)	0.996	1.03 (0.99–1.07)	0.190	1.01 (0.98–1.05)	0.523	0.97 (0.93–1.01)	0.196
No. hours physician/unit	1.02 (0.97–1.07)	0.490	1.01 (0.95–1.07)	0.764	1.01 (0.97–1.06)	0.591	0.98 (0.93–1.03)	0.449	1.00 (0.95–1.06)	0.957

Bold text: statistically significant results. *GMHR* General Medical Health Rating Scale, *PSMS* Physical Self-Maintenance Scale, *CDR sob* Clinical Dementia Rating scale sum of boxes, *CSDD* Cornell Scale for Depression in Dementia, *NPI* Neuropsychiatric Inventory, *QUALID* Quality of Life in Late-Stage Dementia. NPI-subsyndromes are calculated as the sum the following items: NPI-Agitation = Agitation + Disinhibition + Irritability, NPI-Psychosis = Delusions + Hallucinations, NPI-Affective = Depression + Anxiety

^a^ Multiple model; N = 1853(N = 476 at BL, N = 362 at 6m, N = 307 at 12m, N = 241 at 18m, N = 195 at 24m, N = 148 at 30m, N = 124 at 36m)

^**b**^ Regression coefficient (SE) reported because of interaction

Higher scores of CDR sum of boxes were associated with lower odds of prescribing sedatives and hypnotics (OR = 0.89, 95%CI:0.85–0.94, *p* < 0.001).

Being female, higher CSDD score, and NPI-affective subsyndrome score were significantly associated with higher odds of prescribing antidepressants (OR = 2.09, 95%CI:1,26–3.47, *p* = 0.005; OR = 1.05, 95%CI:1.00–1.10, *p* = 0.045 and OR = 1.09, 95%CI:1.04–1.14, *p* < 0.001, respectively). Older age was associated with lower odds of prescribing antidepressants (OR = 0.93, 95%CI:0.90–0.97, p < 0.001).

Younger age and higher NPI-psychosis subsyndrome score were significantly associated with higher odds of prescribing combined typical and atypical antipsychotics (OR = 0.96, 95%CI:0.92–0.99, *p* = 0.023 and OR = 1.11, 95%CI:1.05–1.17, *p* < 0.001, respectively).

Further, we found that with increasing values of NPI-affective subsyndrome score, the odds of prescribing anxiolytics were significantly higher (OR = 1.05, 95%CI:1.01–1.10, *p* = 0.026).

Higher scores on the Charlson Comorbidity Index and NPI-apathy subsyndrome score were associated with lower odds of prescribing antidementia drugs (OR = 0.86, 95%CI:0.75–0.98, *p* = 0.023 and OR = 0.93, 95%CI:0.86–1.00, *p* = 0.039, respectively). Compared to regular or respite and rehabilitation units, patients living in special care units had higher odds of being prescribed antidementia drugs (OR = 1.78, 95%CI:1.09–2.90, *p* = 0.021).

## Discussion

Prevalence of PTD prescription was high overall for the majority of PTD categories, with the highest values for antidepressants; more than 60% of patients received at least one PTD throughout the study period. Our results are in line with previous findings showing how multi-psychotropic drug prescription is associated with severity of NPS [38], symptoms that are a common reason for institutionalization [39], and are persistent in NH patients [2].

In our study we found an increasing prevalence of antidepressants prescription, especially during the first six months after admission. Physicians might in fact promptly identify depression symptoms following NH admission, leading to an appropriate treatment and thereby lower mortality risk [40]. Antidepressants might also be frequently prescribed to treat a high level of NH patients whose depression is resistant to usual treatment with antidepressants, or with a wider indication to treat mood symptoms, such as anxiety and agitation, and not specifically depression [41].

Our study showed that among patients with dementia, up to 29.7% received sedatives/hypnotics and up to 20.8% received antipsychotics. Our findings stand in contrast to a similar study conducted in the USA, presenting a higher prevalence of antipsychotics prescription (28%) and a much lower prevalence of sedatives/hypnotics prescription (2%) [42]. Previous research has also shown a wide discrepancy in the prevalence of sedatives and hypnotics prescriptions in NHs [25, 28]. This difference might have several explanations. A low prevalence of sedatives/hypnotics prescription might be compensated by a higher need to prescribe other medications with sedative effects, such as antipsychotics. On the other hand, sedation is a side effect of antipsychotics, making the use of sedatives/hypnotics less needed. Other factors such as nurses’ distress related to NPS [8, 9], nurse/patient ratios [43], and differences in organizational culture can influence prescriptions of PTDs [44].

In our findings, the prevalence of antipsychotics prescription among people with dementia ranged between 11.7% and 20.7%, results that are higher than data from the UK (8.9%–9.2%) [45], lower than data from Switzerland (36.7%–47.3%) [27], but comparable with data from the USA (14.3%) [46]. A recent Canadian review summarized how both typical and atypical antipsychotics are associated with a higher mortality risk, although this risk is more unclear for atypical antipsychotics compared to typical ones [47]. Antipsychotics prescription has decreased in Norwegian NHs since 2004 [21], and our results confirm that the trend continues. This is probably due to the increases in warnings health authorities have given to limit the use of antipsychotics in people with dementia. It is reassuring that with increasing age, our study showed that the odds of prescribing antipsychotics decreased, as antipsychotics use is associated with a higher risk of adverse effects in older adults [48].

For every PTD category, we found the highest incidence rates between BL and 6m, with the highest values for antipsychotics. NPS are often a reason for NH admission [39], leading physicians to prioritize a pharmacological approach and quickly treat NPS. However, the high level of NPS during the first months might occur because patients need time to familiarize themselves with a new environment, and non-pharmacological approaches should be considered first. Deprescribing rates were relatively stable yet low during the follow-up period. Although caution should be applied while interpreting our results, stable deprescribing rates might still show that there is a focus on a regular medication review, trying to avoid unnecessary prescriptions over time.

Besides an expected significant association between depression, affective symptoms, and odds of being prescribed antidepressants, our study showed that patients with a higher level of affective symptoms had higher odds of being prescribed anxiolytics. This result is comparable with a recent cross-sectional study from the USA [42]. Anxiety is a common symptom of depression, which might be treated with anxiolytics as adjuvants, together with antidepressants.

We found a correlation between lower odds of being prescribed sedatives and hypnotics and increased severity of dementia measured with CDR sum of boxes. Norwegian guidelines do not recommend people with dementia be prescribed sedatives or hypnotics [5], and our findings show a possible caution in prescribing sedatives and hypnotics for people with severe dementia. However, our results still show an alarmingly high prevalence of sedatives and hypnotics prescription during the duration of the study.

When modelling for the odds of prescribing sedatives and hypnotics, the only interaction found was between CSDD score and time. CSDD scores > 8 were associated with higher odds of prescribing sedatives and hypnotics from BL to 18m, and with lower odds of prescribing sedatives and hypnotics from 18m to 36m. A possible interpretation of these results might be that physicians show a more aggressive approach to treat depression with adjuvants, such as sedatives, during the first months after admission, while sedatives and hypnotics might not be considered to treat depression over time in older adults due to the risk of dependency and other side effects [15].

Antidementia drugs were less likely to be prescribed in patients with higher comorbidity. Antidementia drugs might possibly be avoided in patients with dementia who have high comorbidity and, subsequently, short life expectancy due to the risk of side effects. Another possible explanation might be that a large number of NH residents with psychiatric and somatic comorbidity have a potentially undetected dementia [49], leading physicians not to prescribe antidementia drugs to this group of patients. We found that patients with a higher degree of apathy were less likely to be prescribed antidementia drugs. Apathy might not be considered a symptom to be medicated, and a previous review showed that other behavioral symptoms, rather than apathy, were more sensitive to treatment with anti-dementia drugs [50]. However, a large meta-analysis has recently shown how cholinesterase inhibitors, although effective in treating cognitive symptoms in patients with Alzheimer’s disease, did not improve NPS [51].

Due to the lack of longitudinal NH studies following prescription practices from admission, this study offers new information about PTD prescription over time, particularly its association with clinical and environmental factors. The short intervals between assessment points give a more accurate overview of prescription trends. The study used standardized and validated assessment tools, making it easy to compare results with other international studies.

This study has some limitations. Dementia status was primarily assessed according to BL data, but it was not assessed at the succeeding assessment points. Hence, we did not include dementia status as a covariate in the regression analysis. However, CDR was used as covariate and as indicator of cognitive impairment, and most participants in this study already had dementia at BL, making the dementia subgroup predominant. Inconsistencies might have been present during data collection, due to the high number of NH staff who assessed the participants, despite the use of standardized tools. However, the staff received extensive training prior to the study. About 50% of the eligible patients from the 47 included NHs did not participate in the study for different reasons, listed in detail in a previous paper [31]. Some participants dropped out or died during the follow-up period, resulting in a drastically reduced number of participants remaining at the later assessment points, and in this way affecting the power of the study. Due to reduced power, some potentially significant associations in multiple models might have been lost. By using a generalized linear mixed model to analyze the data, we minimized, to some extent, the bias due to missing data. However, a high drop-out rate might have introduced attrition bias, making difficult to distinguish the effects of covariates on the use of PTDs and attrition. We advise therefore a cautious interpretation of our data, as attrition bias may change the interpretation of the results from non-significant to significant [52]. The participants were recruited from different NHs. We did not present the distribution of the participants for each included NH. However, we considered the size of the ward in which each participant was living, and included this information in the regression analysis. Data about medication “as needed” were unfortunately not recorded during data collection [31]. Even if many PTDs, i.e., antidepressants and antipsychotics, are commonly prescribed as regular medication, it is common in a clinical setting to prescribe sedatives / hypnotics and anxiolytics as needed. Thus, our study might present an underrepresentation for these drugs, and our results might underestimate the use of some PTD categories over time.

## Conclusions

PTDs are extensively prescribed in NHs, already from admission, and there is an increasing trend of prescribing antidepressants and antipsychotics over time. Every PTD category had its highest incidence rate the first six months after NH admission. Higher age seems to decrease the risk of being prescribed antipsychotics, and severity of dementia seems to decrease the odds of being prescribed sedatives and hypnotics. Particular attention should be given to frequently assessing treatment with PTDs in NH patients to avoid prolonged and excessive exposure to these medications.

## Data Availability

The datasets generated and/or analyzed during the current study are not publicly available due to the sensitive nature of the data, but are available from the corresponding author on reasonable request and after approval by the Regional Committee for Medical and Health Research Ethics.

## Abbreviations

PTD: Psychotropic drug
NH: Nursing home
NPS: Neuropsychiatric symptoms
BL: Baseline
6m, 12m, 18m, 24m, 30m, 36m: 6-, 12-, 18-, 24-, 30-, 36-months follow-up, respectively
CI: Confidence interval
SE: Standard error
OR: Odds ratio

## References

[1] Helvik AS, Engedal K, Benth JS, Selbaek G (2015). Prevalence and severity of dementia in nursing home residents. Dement Geriatr Cogn Disord.

[2] Helvik AS, Selbaek G, Saltyte Benth J, Roen I, Bergh S (2018). The course of neuropsychiatric symptoms in nursing home residents from admission to 30-month follow-up. PLoS One.

[3] Kales HC, Gitlin LN, Lyketsos CG (2015). Assessment and management of behavioral and psychological symptoms of dementia. BMJ (Clinical research ed).

[4] Brimelow RE, Wollin JA, Byrne GJ, Dissanayaka NN (2019). Prescribing of psychotropic drugs and indicators for use in residential aged care and residents with dementia. Int Psychogeriatr.

[5] Helsedirektoratet (2017). Nasjonal faglig retningslinje om demens.

[6] Dudas R, Malouf R, McCleery J, Dening T (2018). Antidepressants for treating depression in dementia. Cochrane Database Syst Rev.

[7] Smeets CHW, Zuidema SU, Hulshof TA, Smalbrugge M, Gerritsen DL, Koopmans R (2018). Efficacy of antipsychotics in dementia depended on the definition of patients and outcomes: a meta-epidemiological study. J Clin Epidemiol.

[8] Zuidema SU, de Jonghe JF, Verhey FR, Koopmans RT (2011). Psychotropic drug prescription in nursing home patients with dementia: influence of environmental correlates and staff distress on physicians’ prescription behavior. Int Psychogeriatr.

[9] Smeets CHW, Gerritsen DL, Zuidema SU, Teerenstra S, van der Spek K, Smalbrugge M, et al. Psychotropic drug prescription for nursing home residents with dementia: prevalence and associations with non-resident-related factors. Aging Ment Health. 2017:1–9. 10.1080/13607863.2017.1348469 28726490

[10] Lemay CA, Mazor KM, Field TS, Donovan J, Kanaan A, Briesacher BA, Foy S, Harrold LR, Gurwitz JH, Tjia J (2013). Knowledge of and perceived need for evidence-based education about antipsychotic medications among nursing home leadership and staff. J Am Med Dir Assoc.

[11] Shaw C, Williams KN, Perkhounkova Y (2018). Educating nursing home staff in dementia sensitive communication: impact on antipsychotic medication use. J Am Med Dir Assoc.

[12] Janus SI, van Manen JG, IJzerman MJ, Bisseling M, Drossaert CH, Zuidema SU (2017). Determinants of the nurses’ and nursing assistants’ request for antipsychotics for people with dementia. Int Psychogeriatr.

[13] Janus SIM, van Manen JG, Zuidema SU, Snijder C, Drossaert CHC, Ijzerman MJ (2018). Reasons for (not) discontinuing antipsychotics in dementia. Psychogeriatrics..

[14] da Costa FA, Periquito C, Carneiro MC, Oliveira P, Fernandes AI, Cavaco-Silva P (2016). Potentially inappropriate medications in a sample of Portuguese nursing home residents: does the choice of screening tools matter?. Int J Clin Pharm.

[15] Nyborg G, Straand J, Klovning A, Brekke M (2015). The Norwegian general practice--nursing home criteria (NORGEP-NH) for potentially inappropriate medication use: a web-based Delphi study. Scand J Prim Health Care.

[16] Mihanović M, Bodor D, Kezić S, Restek-Petrović B, Silić A (2009). Differential diagnosis of psychotropic side effects and symptoms and signs of psychiatric disorders. Psychiatr Danub.

[17] El Haddad K, de Souto BP, de Mazieres CL, Rolland Y (2020). Effect of a geriatric intervention aiming to improve polypharmacy in nursing homes. Eur Geriatr Med.

[18] Hajjar ER, Cafiero AC, Hanlon JT (2007). Polypharmacy in elderly patients. Am J Geriatr Pharmacother.

[19] Gustafsson M, Sandman PO, Karlsson S, Gustafson Y, Lovheim H (2013). Association between behavioral and psychological symptoms and psychotropic drug use among old people with cognitive impairment living in geriatric care settings. Int Psychogeriatr.

[20] Maclagan LC, Maxwell CJ, Gandhi S, Guan J, Bell CM, Hogan DB (2017). Frailty and Potentially Inappropriate Medication Use at Nursing Home Transition. J Am Geriatr Soc.

[21] Selbaek G, Janus SIM, Bergh S, Engedal K, Ruths S, Helvik AS, et al. Change in psychotropic drug use in Norwegian nursing homes between 2004 and 2011. Int Psychogeriatr. 2017:1–10. 10.1017/s1041610217001788. 10.1017/S1041610217001788 28988552

[22] Helvik AS, Saltyte Benth J, Wu B, Engedal K, Selbaek G (2017). Persistent use of psychotropic drugs in nursing home residents in Norway. BMC Geriatr.

[23] Scocco P, Rapattoni M, Fantoni G (2006). Nursing home institutionalization: a source of eustress or distress for the elderly?. Int J Geriatr Psychiatry..

[24] Nygaard HA (2001). Chronisity of drug treatment in nursing home residents. Tidsskr Nor Lægeforen 2001.

[25] O'Connor DW, Griffith J, McSweeney K (2010). Changes to psychotropic medications in the six months after admission to nursing homes in Melbourne, Australia. Intern Psychogeriatr.

[26] Bakken MS, Ranhoff AH, Engeland A, Ruths S (2012). Inappropriate prescribing for older people admitted to an intermediate-care nursing home unit and hospital wards. Scand J Prim Health Care.

[27] Lustenberger I, Schupbach B, von Gunten A, Mosimann U (2011). Psychotropic medication use in Swiss nursing homes. Swiss Med Wkly.

[28] Ivanova I, Wauters M, Stichele RV, Christiaens T, De Wolf J, Dilles T (2018). Medication use in a cohort of newly admitted nursing home residents (ageing@NH) in relation to evolving physical and mental health. Arch Gerontol Geriatr.

[29] Callegari E, Benth J, Selbæk G, Grønnerød C, Bergh S. Does Psychotropic Drug Prescription Change in Nursing Home Patients the First 6 Months After Admission? J Am Med Dir Assoc. 2020;22(1):101–8.e1. 10.1016/j.jamda.2020.08.034. 10.1016/j.jamda.2020.08.034 33077352

[30] Cummings JL, Mega M, Gray K, Rosenberg-Thompson S, Carusi DA, Gornbein J (1994). The neuropsychiatric inventory: comprehensive assessment of psychopathology in dementia. Neurology..

[31] Roen I, Selbaek G, Kirkevold O, Engedal K, Testad I, Bergh S (2017). Resourse use and disease Couse in dementia - nursing home (REDIC-NH), a longitudinal cohort study; design and patient characteristics at admission to Norwegian nursing homes. BMC Health Serv Res.

[32] Morris JC (1993). The clinical dementia rating (CDR): current version and scoring rules. Neurology..

[33] Lawton MP, Brody EM (1969). Assessment of older people: self-maintaining and instrumental activities of daily living. The Gerontologist.

[34] Alexopoulos GS, Abrams RC, Young RC, Shamoian CA (1988). Cornell scale for depression in dementia. Biol Psychiatry.

[35] Lyketsos CG, Galik E, Steele C, Steinberg M, Rosenblatt A, Warren A, Sheppard JM, Baker A, Brandt J (1999). The general medical health rating: a bedside global rating of medical comorbidity in patients with dementia. J Am Geriatr Soc.

[36] Charlson ME, Pompei P, Ales KL, MacKenzie CR (1987). A new method of classifying prognostic comorbidity in longitudinal studies: development and validation. J Chronic Dis.

[37] Weiner MF, Martin-Cook K, Svetlik DA, Saine K, Foster B, Fontaine CS (2000). The quality of life in late-stage dementia (QUALID) scale. J Am Med Dir Assoc.

[38] Gulla C, Selbaek G, Flo E, Kjome R, Kirkevold O, Husebo BS (2016). Multi-psychotropic drug prescription and the association to neuropsychiatric symptoms in three Norwegian nursing home cohorts between 2004 and 2011. BMC Geriatr.

[39] Afram B, Stephan A, Verbeek H, Bleijlevens MH, Suhonen R, Sutcliffe C, Raamat K, Cabrera E, Soto ME, Hallberg IR, Meyer G, Hamers JP, RightTimePlaceCare Consortium (2014). Reasons for institutionalization of people with dementia: informal caregiver reports from 8 European countries. J Am Med Dir Assoc.

[40] Damian J, Pastor-Barriuso R, Valderrama-Gama E, de Pedro-Cuesta J (2017). Association of detected depression and undetected depressive symptoms with long-term mortality in a cohort of institutionalised older people. Epidemiol Psychiatr Sci.

[41] Barca ML, Engedal K, Laks J, Selbaek G (2010). A 12 months follow-up study of depression among nursing-home patients in Norway. J Affect Disord.

[42] Resnick B, Kolanowski A, Van Haitsma K, Galik E, Boltz M, Ellis J, et al. Current Psychotropic Medication Use and Contributing Factors Among Nursing Home Residents With Cognitive Impairment. Clin Nurs Res. 2019;30(1):59–69. 10.1177/1054773819838678. 10.1177/1054773819838678 PMC6776729 30943786

[43] Kim H, Whall AL (2006). Factors associated with psychotropic drug usage among nursing home residents with dementia. Nurs Res.

[44] Tjia J, Gurwitz JH, Briesacher BA (2012). Challenge of changing nursing home prescribing culture. Am J Geriatr Pharmacother.

[45] Ballard C, Corbett A, Orrell M, Williams G, Moniz-Cook E, Romeo R, Woods B, Garrod L, Testad I, Woodward-Carlton B, Wenborn J, Knapp M, Fossey J (2018). Impact of person-centred care training and person-centred activities on quality of life, agitation, and antipsychotic use in people with dementia living in nursing homes: a cluster-randomised controlled trial. PLoS Med.

[46] The U.S. Centers for Medicare & Medicaid Services (2020). National Partnership to Improve Dementia Care in Nursing Homes: Antipsychotic Medication Use Data Report.

[47] Randle JM, Heckman G, Oremus M, Ho J (2019). Intermittent antipsychotic medication and mortality in institutionalized older adults: a scoping review. Int J Geriatr Psychiatry.

[48] Schneider LS, Tariot PN, Dagerman KS, Davis SM, Hsiao JK, Ismail MS, Lebowitz BD, Lyketsos CG, Ryan JM, Stroup TS, Sultzer DL, Weintraub D, Lieberman JA (2006). Effectiveness of atypical antipsychotic drugs in patients with Alzheimer’s disease. N Engl J Med.

[49] Bartfay E, Bartfay WJ, Gorey KM (2013). Prevalence and correlates of potentially undetected dementia among residents of institutional care facilities in Ontario, Canada, 2009-2011. Int J Geriatr Psychiatry..

[50] Molino I, Colucci L, Fasanaro AM, Traini E, Amenta F (2013). Efficacy of memantine, donepezil, or their association in moderate-severe Alzheimer's disease: a review of clinical trials. ScientificWorldJournal..

[51] Blanco-Silvente L, Castells X, Saez M, Barceló MA, Garre-Olmo J, Vilalta-Franch J, Capellà D (2017). Discontinuation, efficacy, and safety of cholinesterase inhibitors for Alzheimer’s disease: a Meta-analysis and Meta-regression of 43 randomized clinical trials enrolling 16 106 patients. Int J Neuropsychopharmacol.

[52] Akl EA, Briel M, You JJ, Sun X, Johnston BC, Busse JW, Mulla S, Lamontagne F, Bassler D, Vera C, Alshurafa M, Katsios CM, Zhou Q, Cukierman-Yaffe T, Gangji A, Mills EJ, Walter SD, Cook DJ, Schunemann HJ, Altman DG, Guyatt GH (2012). Potential impact on estimated treatment effects of information lost to follow-up in randomised controlled trials (LOST-IT): systematic review. BMJ : British Medical Journal.

