# Correction: Do prescription rates of psychotropic drugs change over three years from nursing home admission?

**DOI:** 10.1186/s12877-022-03486-6

**Published:** 2022-12-22

**Authors:** Enrico Callegari, Jūratė Šaltytė Benth, Geir Selbæk, Cato Grønnerød, Sverre Bergh

**Affiliations:** 1 Østfold Hospital Trust, Sykehuset Østfold HF, postboks 300, 1714 Grålum, Norway; 2 Faculty of Medicine, University of Oslo, Oslo, Norway; 3 Institute of Clinical Medicine, University of Oslo, Campus Ahus, Oslo, Norway; 4 Research Centre for Age-related Functional Decline and Diseases, Innlandet Hospital Trust, Ottestad, Norway; 5 Health Services Research Unit, Akershus University Hospital, Lørenskog, Norway; 6 Norwegian National Advisory Unit on Ageing and Health, Vestfold Hospital Trust, Tønsberg, Norway; 7 Department of Geriatric Medicine, Oslo University Hospital, Oslo, Norway; 8 Faculty of Social Sciences, Department of Psychology, University of Oslo, Oslo, Norway

**BMC Geriatrics (2021) 21:496**

10.1186/s12877-021-02437-x

After publication of this article [1], the authors detected an error in the analyses: the prevalence for antipsychotics in people with dementia should be between 11.7% and 20.8%, and not between 11.7% and 20.7%, as stated in the original article. The reported descriptive incidence and deprescribing rates for psychotropic drugs were also revised. This caused corrections in the abstract, Table 2, Fig. 3 and the Discussion section to be made.

The original article [1] has been corrected.
